# The MADS-Box Gene *MdDAM1* Controls Growth Cessation and Bud Dormancy in Apple

**DOI:** 10.3389/fpls.2020.01003

**Published:** 2020-07-07

**Authors:** Mirko Moser, Elisa Asquini, Giulia Valentina Miolli, Kathleen Weigl, Magda-Viola Hanke, Henryk Flachowsky, Azeddine Si-Ammour

**Affiliations:** ^1^Research and Innovation Centre, Fondazione Edmund Mach (FEM), San Michele all’Adige (TN), Italy; ^2^Julius Kühn-Institut (JKI), Federal Research Centre for Cultivated Plants, Institute for Breeding Research on Fruit Crops, Dresden, Germany

**Keywords:** chilling temperature, cold, bud dormancy, growth cessation, MADS-box gene, *Malus × domestica* (apple)

## Abstract

Apple trees require a long exposure to chilling temperature during winter to acquire competency to flower and grow in the following spring. Climate change or adverse meteorological conditions can impair release of dormancy and delay bud break, hence jeopardizing fruit production and causing substantial economic losses. In order to characterize the molecular mechanisms controlling bud dormancy in apple we focused our work on the MADS-box transcription factor gene *MdDAM1*. We show that *MdDAM1* silencing is required for the release of dormancy and bud break in spring. *MdDAM1* transcript levels are drastically reduced in the low-chill varieties ‘Anna’ and ‘Dorsett Golden’ compared to ‘Golden Delicious’ corroborating its role as a key genetic factor controlling the release of bud dormancy in *Malus* species. The functional characterization of *MdDAM1* using RNA silencing resulted in trees unable to cease growth in winter and that displayed an evergrowing, or evergreen, phenotype several years after transgenesis. These trees lost their capacity to enter in dormancy and produced leaves and shoots regardless of the season. A transcriptome study revealed that apple evergrowing lines are a genocopy of ‘Golden Delicious’ trees at the onset of the bud break with the significant gene repression of the related MADS-box gene *MdDAM4* as a major feature. We provide the first functional evidence that MADS-box transcriptional factors are key regulators of bud dormancy in pome fruit trees and demonstrate that their silencing results in a defect of growth cessation in autumn. Our findings will help producing low-chill apple variants from the elite commercial cultivars that will withstand climate change.

## Introduction

Apple (*Malus × domestica* Borkh.) is an economically important fruit tree cultivated in temperate regions around the globe. When grown in the Northern Hemisphere, apple trees usually enter in dormancy at the beginning of autumn. During this season apple buds containing the meristems first cease to grow followed by the establishment of the dormancy and the acquisition of cold hardiness prior to winter (Maurya and Bhalerao, 2017). Apple tree growth cessation and bud dormancy processes in autumn are tightly linked and are induced solely by cold and not by photoperiod (Heide and Prestrud, 2005). After an arrest during winter, vegetative growth resumes in spring followed by the appearance of flowers and leaves and the development of new shoots. Fruitlets are rapidly formed in spring and fruits grow and ripen during summer. During this period, terminal and lateral buds are formed and will gradually enter in dormancy. Three different consecutive types of dormancy, overlapping from late summer to early spring, can be distinguished: paradormancy, endodormancy, and ecodormancy (Lang, 1987). The paradormant stage starts when bud growth is suppressed by internal factors within the plant but outside the bud structure by a mechanism called correlative inhibition. Buds are considered endodormant when a signal within the bud triggers deep dormancy that occurs typically during autumn and winter. At this stage, development is suppressed even in growth conducive conditions. It is usually referred to endodormancy when speaking about dormancy. During this stage the bud accumulates exposure time to chilling, which is required to break endodormancy and to restore the competency to grow. After endodormancy has been completed the plant still remains dormant. This period is called ecodormancy since buds are usually exposed to harsh environmental conditions unfavorable for growth, such as temperatures below 10°C (Lang, 1987). Once the ambient temperature increases, the bud breaks quickly, followed by a rapid growth of leaf and flower structures.

Most of the apple elite cultivars cultivated in temperate areas require long exposure to chilling temperatures during dormancy to acquire the competence to flower in spring and this is referred as the chilling requirement (CR). The duration of exposure to cold, or more precisely, chilling temperatures, is expressed either as chilling units (CU) or chilling hours (CH). For instance, apple elite varieties grown in Northern Europe with cold winters such as ‘Golden Delicious’, ‘Pinova’ or ‘Gala’ require between 500 to 800 CH and flower (Rea and Eccel, 2006; Guak and Neilsen, 2013). In order to grow apples in warmer countries breeders developed cultivars with low CR such as ‘Anna’ and ‘Dorsett Golden’ that requires less than 300 CH to flower (Hauagge and Cummins, 1991). Diverse environmental factors, including global warming, have a direct influence on bud phenology. The consequences on fruit production can be different according to the geographical location. For instance, higher temperatures in Northern Europe forces the bud to break earlier hence increasing the risk of frost damage to the newly formed green tissues. In contrast, climate warming in Southern Europe affect the chilling requirements with a direct impact on bud break and flowering periods (Legave et al., 2013). The use of toxic chemicals such as hydrogen cyanamide is often the last resort for fruit growers to artificially break dormancy but with adverse effects on human health and on the environment (Sheshadri et al., 2011).

The molecular mechanisms controlling flowering in response to chilling temperatures have been extensively studied in the annual winter plant *Arabidopsis thaliana* (Kim et al., 2009). The MADS-box gene *FLOWERING LOCUS C* (*FLC)* is the key regulator of the vernalization pathway in Arabidopsis (Michaels and Amasino, 1999). *FLC* acts as a floral repressor and its transcription is gradually repressed during winter in an epigenetic manner by an increase of tri-methylation at Lysine 27 on histone H3 (H3K27me3). A similar cold-dependent epigenetic repression of MADS-box genes has been described in the perennial plant *Arabis alpina* as well as in the evolutionary distant fruit trees *Prunus persica* (peach) and *Pyrus pyrifolia* (Japanese pear). An increase in H3K27me3 marks were measured on *AaPEP1* chromatin in *A. alpina* and on the Dormancy-Associated MADS-box (DAM) genes *PpDAM6* and *PpMADS13-1* loci in peach and Japanese pear, respectively (Wang et al., 2009; Leida et al., 2012; Saito et al., 2015). Although bud dormancy in trees and vernalization in annual plants have the epigenetic regulation of MADS-box genes triggered by long exposure to cold in common, the physiological mechanisms leading to bud break and flowering are drastically different (Chouard, 1960).

Recent studies attempted to make the link between MADS-box genes and dormancy in apple and several gene members that belong to the *StMADS11* specific sub-clade were proposed to control dormancy and bud break. These genes turned out to have more similarities with the Arabidopsis *AGAMOUS-like* 24 (*AGL24)* and *short vegetative phase* (*SVP* or *AGL22)* genes than with *FLC* (Velasco et al., 2010). Among the nine apple *AGL24*-like or *SVP*-like genes included in this sub-clade, four are orthologues of the six dormancy-associated (*PpDAM1 to 6*) genes that control dormancy in peach. They were subsequently named *MdDAM1* (MDP0000322567), *MdDAM2* (MDP0000259294), *MdDAM3* (MDP0000527190), and *MdDAM4* (MDP0000232313). The five other genes have the nomenclature *MdJa* (MDP0000233948), *MdJb* (MDP0000209705) and *SVP-like/DAM-like* (MDP0000259296, MDP0000285270, MDP0000255146) (Porto et al., 2016). Their expression analyses during bud dormancy time course revealed that only *MdDAM1* and *MdDAM4*, and to a less extent *MdDAM3*, have a gene profile that reacts to seasonal changes that is quite similar to the one of *AtFLC* and *AaPEP1*, namely a high expression in autumn followed by a gradual repression and a total silencing in spring (Mimida et al., 2015; Porto et al., 2015; Wisniewski et al., 2015; Kumar et al., 2016). In contrast, *MdDAM2*, *MdJa*, and *MdJb* expression did not fluctuate during the different seasons. Consistent with that observation, ectopic expression of *MdDAM3* and *MdJa* in apple resulted in delayed bud break and a change in tree architecture but not in a change in bud dormancy or growth cessation in autumn (Wu et al., 2017).

In this work, we rather focused our study on the functional characterization of *MdDAM1* that showed an interesting gene profile during seasonal changes suggesting a role in bud dormancy and growth cessation in autumn. We expressed it and silenced it constitutively in apple and we report that it has a crucial role in growth cessation. Additionally we performed a transcriptome analysis of the transgenic trees and show that *MdDAM1* might control a regulatory network including the second apple DAM gene *MdDAM4* and several gene families with interesting physiological functions.

Prediction models unanimously converge to the conclusion that winters will become warmers due to climate change. This catastrophic event will have a negative impact on bud dormancy and bud break processes of deciduous fruit trees such as apple (Atkinson et al., 2013). Therefore, understanding the molecular mechanisms governing apple bud dormancy and growth cessation in apple is crucial to enable the breeding of varieties with lower chilling requirements and we show in this study that manipulating *MdDAM1* gene expression is one research direction to follow.

## Material and Methods

### Plant Material and Environmental Parameters

The *Malus* × *domestica* Borkh. cultivars ‘Golden Delicious’ clone B, ‘Anna’ (Geneva accession No PI 280400), ‘Dorsett Golden’ (Geneva accession No PI 589913) grafted on M9 rootstock were used in this study. Terminal dormant buds were harvested from ten year-old trees growing in the “Giaroni” orchard in San Michele all’Adige in Italy (latitude 46.181539°, longitude 11.119877°). Buds were collected each year from 2011 to 2019 on the 15th of October, 15th of November, 15th of December, 15th of January, 15th of February and the 8th of March which corresponds to the bud break of ‘Golden Delicious’. The bud break in ‘Anna’ and ‘Dorsett Golden’ occurred already on the 15^th^ of February. Temperature and day length measurements were obtained from the meteorological station located directly in the orchard.

### Apple Transformation and Molecular Characterization of Transgenic Lines

Transformation of the apple cultivar ‘Pinova’ and the subsequent regeneration and propagation steps were performed as previously described (Flachowsky et al., 2007). The plasmid vector pP35S:*MdDAM1* was constructed by DNA Cloning Service (Hamburg, Germany) and transferred to the *Agrobacterium tumefaciens* strain EHA105. Transgenic plants and non-transgenic control (NT) ‘Pinova’ were micro-grafted onto ‘Golden Delicious’ seedlings as previously described (Vanblaere et al., 2011). For cultivation and phenotypic evaluation, all plants were grown in the glasshouse from May to August without additional light. Detection of transgenic DNA sequences was done by PCR on genomic DNA extracted from 1 g leaf tissue of *in vitro* plants using the DNeasy Plant Mini Kit (Qiagen, Hilden, Germany). Standard PCR using the primers listed in **Table S1** was performed using the Dream-Taq DNA polymerase (Fisher Scientific, Schwerte, Germany) following the manufacturer’s recommendations. The Southern blot was prepared by first extracting genomic DNA from young leaves of *in vitro* grown shoots using a modified CTAB protocol (Saghai-Maroof et al., 1984). Ten micrograms of genomic DNA was digested with *Bam*HI (Fisher Scientific, Schwerte, Germany) and blotted onto a positively charged nylon membrane (Roche Deutschland Holding GmbH, Mannheim, Germany). The membrane was hybridized with a DIG-labeled nptII probe generated by PCR using the primers nptII_F and nptII_R (**Table S1**). The signal was detected using the anti-DIG-AP (Roche Deutschland Holding GmbH) and ECF substrate (Amersham Biosciences Europe GmbH, Freiburg, Germany) on a ChemiDoc XRS^+^ System (Bio-Rad Laboratories GmbH).

### Phenotypic Evaluation of Dormancy Switch

To determine the switch from vegetative growth to dormancy nine to fifteen plants per genotype (transgenic and NT control) were grown in a glasshouse until the middle of August. At this date, all shoots were pruned at 20 cm above soil level to obtain uniform plants. Starting from this day optimal growth conditions with 16 h of light and 8 h of darkness and temperature above 20°C were applied to facilitate the shoot re-growth. On the 21st of October, the application of extra light was stopped and temperatures made similar to those in the orchard for dormancy induction. To avoid frost damage an ‘artificial winter’ program was applied as previously described (Flachowsky et al., 2011). The developing shoot re-growth was evaluated weekly for a total of 27 weeks starting from the 11th of September (three weeks after pruning) using a dormancy switch index (DSI) scale from 1 to 5 described as follows: 1 = Shoot is actively growing. Young leaves at the top of the shoot are light green whereas older leaves are dark green colored. 2 = Shoot growth is decreasing. Young leaves at the top are still light green colored. 3 = Shoot growth is almost stopped. Young leaves have nearly the same color than leaves of the remaining shoot. 4 = Shoot growth is completely stopped. The terminal bud at the shoot apex starts to enter dormancy. 5 = the terminal buds entered in deep dormancy and the growth ceased completely. Significance of the switch to dormancy relative to the NT control was determined using Student’s t-test.

### RNA Analysis and qRT-PCR

Terminal buds were harvested and the bud scales were quickly removed. The leaves and the green tissues from buds were flash frozen in liquid nitrogen and homogenized. Total RNA was extracted using the Plant spectrum kit (Sigma-Aldrich, St. Louis, MO, USA) followed by on-column DNase digestion with DNase I (Sigma-Aldrich, St. Louis, MO, USA) according to the manufacturer’s instruction. RNA integrity was verified by capillary electrophoresis (Agilent Technologies, Santa Clara, CA, USA) and samples with suitable RIN (> 8) were analyzed. The relative expression level of *MdDAM1* and *MdDAM4* was checked by qRT-PCR using the primers *MdDAM1*_Fq and *MdDAM1*_Rq for *MdDAM1* and *MdDAM4*_Fq and *MdDAM4*_Rq for *MdDAM4* (**Table S1**) with the Maxima qPCR Master Mix (Thermo Scientific) on a CFX96 Touch™ Real-Time PCR Detection System (Bio-Rad Laboratories, Munich, Germany) as previously described (Flachowsky et al., 2012). Fold expression of *MdDAM1* and *MdDAM4* in transgenic lines relative to the NT control plants was normalized to ELONGATION FACTOR 1 alpha (EF1α, MDP0000294265) reference gene using the primers indicated in **Table S1** (Poupard et al., 2003; Wisniewski et al., 2015). *MdEF1α* (MDP0000294265), *MdACTIN*/MDP0000912745 (Li and Yuan, 2008), and *Md_4592:1:a* (Botton et al., 2011) were found to be the most stably expressed reference genes in apple buds in the RNA-seq data produced during this study using the procedure described in Zhou et al. (2017). The stability of *MdEF1α* (MDP0000294265) expression was found to be superior to *MdACTIN* and *Md_4592:1:a* using NormFinder (Andersen et al., 2004) and was therefore used alone as a reference gene for all qRT-PCR experiments performed in this work. The results were analyzed using the comparative Ct method (Pfaffl, 2001) on three biological replicates composed of a mix of 30 terminal buds each sampled from three to five different trees. The qRT-PCR was performed using three technical replicates. The significance levels of *MdDAM1* and *MdDAM4* expression relative to the NT control were determined using Student’s t-test

### RNA-Seq and sRNA-Seq Analysis

Three independent biological replicates, consisting of five to ten terminal buds harvested from three different individual trees, were taken at each time point to build the RNA- and sRNA-seq libraries. Total RNA was extracted from these buds as described above whereas sRNA was extracted using the mirPremier microRNA kit (Sigma-Aldrich, Italy) following the manufacturer’s instructions. For each time point The RNA-Seq and sRNA-Seq libraries were built using the TruSeq RNA and TruSeq Small RNA Library Prep kits (Illumina, Italy), respectively, following the manufacturer’s protocol. The libraries were sequenced in multiplexing (maximum of six libraries per lane) using the single read 1 × 100 bp mode on a HiSeq2000 platform (Illumina, Switzerland) at Fasteris (Geneva, Switzerland) and at the LaBSSAH facility (Trento, Italy, www.labssah.eu). The sRNA libraries were sequenced using a single read 1 × 36 bp mode on an Illumina Miseq platform (Illumina, Italy) at the Fondazione Edmund Mach with a Miseq Reagent Kit v3. To analyze the RNA-seq data the reads were first cleaned from adapter sequences and the residual rRNA sequences were filtered. Abundances of reads unique for each transcript were counted at each time point and reported in a raw abundance matrix. The reads were then subjected to differential expression analysis using DESeq2 with default parameters (Love et al., 2014). Transcripts with an adjusted p-value <0.01 at March were further filtered and only those with an absolute value for the log2 fold change (log2FC) ≥1 were considered as differentially expressed. For each transcript the log2FC values from the pairwise comparison of each time point against “October” time point were retrieved from the DESeq2 calculations. The resulting matrix was used to calculate the distance matrix with the dist function of R (“euclidean” method) followed by the hierarchical clustering carried out with the function hclust (“complete” method). The heatmap showing the gene hierarchical clustering was prepared using the R package heatmap.2 of gplots library. The sRNA-seq data was produced as follows. Truseq small RNA adapters were removed from the raw reads using cutadapt v2.1 (Martin, 2011). The 21–24 nt long reads were selected and aligned against the pP35S:*MdDAM1* T-DNA sequence with bowtie (v1.2.3) keeping only alignments with perfect match (Langmead et al., 2009). The plots of sRNA abundance were produced using “matplotlib” (Hunter, 2007).

## Results

### *MdDAM1* Is Lowly Expressed in Low-Chill Apple Cultivars

**Figure 1:**
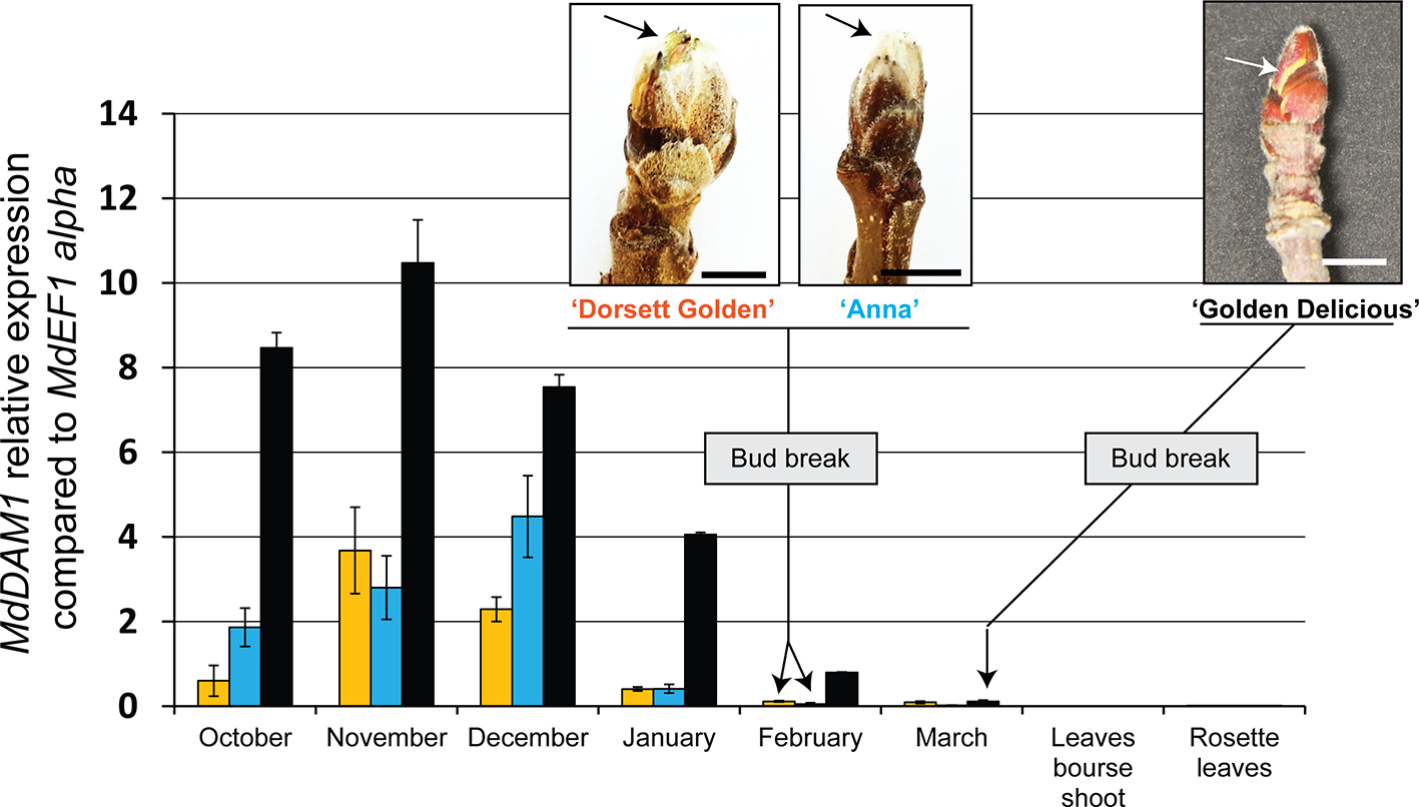
Expression of *MdDAM1* in dormant buds and in leaves. The relative expression values of *MdDAM1* was measured in buds at different time points from the onset of dormancy in October to bud break in March for ‘Golden Delicious’ (black bars) or February for ‘Dorsett Golden’ (orange bars) and ‘Anna’ (blue bars) cultivars. The expression of *MdDAM1* was also studied in leaves of these cultivars after the emergence of flower clusters in spring. *MdDAM1* relative expression compared to the housekeeping gene *MdEF1* alpha was determined by qRT-PCR. Error bars represent standard deviation from three biological replicates. Representative picture at bud break for each cultivar tested are indicated (bar = 0.5 cm). The arrow in each picture indicates the green sectors appearing at bud break.

We first checked the expression of *MdDAM1* in terminal buds during the course of dormancy of the widely cultivated ‘Golden Delicious’ and the low-chill cultivars ‘Dorsett Golden’ and ‘Anna’. The expression of *MdDAM1* was also measured in different type of leaves originating from the buds of these varieties in spring after the emergence of the flower cluster. Gene expression analysis using qRT-PCR shows that *MdDAM1* is undetectable in leaves from the bourse shoots or in those from the rosette surrounding the flower cluster of all cultivar tested (**Figure 1**). In contrast, *MdDAM1* was expressed at high levels in dormant buds of ‘Golden Delicious’ collected in October and November and was gradually repressed during dormancy in winter to be completely silenced in March when the bud breaks and the temperature as well as the day length increase at the onset of spring (**Figure 1**, **Figure S1**). In contrast to ‘Golden Delicious’, the expression of *MdDAM1* is constitutively low during autumn and winter in the low-chill cultivars ‘Dorsett Golden’ and ‘Anna’. *MdDAM1* expression is already silenced in January in both low-chill cultivars two months earlier than in ‘Golden Delicious’. The bud break already occurred at the beginning of February in both low-chill cultivars and the full bloom is on the 31st of March which is roughly three weeks before ‘Golden Delicious’ (**Figure 1**, **Figure S1**). This difference in *MdDAM1* expression between the low-chill cultivars and ‘Golden Delicious’ could be either due to a change in one single nucleotide leading to the substitution of a methionine (M) to valine (V) at position 194 (**Figure S2**), or to other epigenetic factors. Taken all together these results suggest that *MdDAM1* expression seems to be restricted to buds and that its temperature- and time-dependent gene silencing correlates with bud break and release of dormancy in apple. Additionally, *MdDAM1* could serve as a marker for distinguishing low-chill from high-chill cultivars.

### Phenotypical Characterization of the *MdDAM1* Transgenic Lines Reveals a Loss of Dormancy

**Figure 2:**
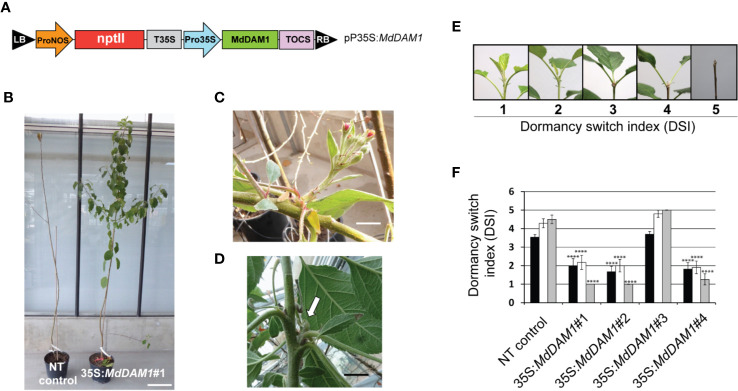
Construct used to express *MdDAM1* in apple and phenotype of the transgenic trees. The T-DNA vector pPro35S:*MdDAM1* from Left Border (LB) to Right Border (RB) used to transform apple consists of a cassette containing a neomycine phosphotranferase gene (*nptII*) under the ProNOS promoter and the coding sequence of *MdDAM1* under the Pro35S promoter. The terminator T35S and TOCS were used for *nptII* and *MdDAM1*, respectively **(A)**. Picture taken end of November of the second year after transformation and showing that the transgenic line 35S:*MdDAM1*#1, (right) is unable to enter dormancy and display more lateral branching compared to the non-transformed control (NT control) that already entered in dormancy and consists on a main branch containing only one single side shoot **(B)** (bar = 30 cm). The transgenic tree line 35S:*MdDAM1*#1 produced flower clusters **(C)** and buds **(D)** in the third winter and throughout all the following years and always stayed green regardless of the season (bar = 2 cm in **C**, **D**). Representative pictures of the terminal apex of apple trees at different stages of dormancy. A dormancy switch index (DSI) of 1 indicates a constant vegetative growth whereas a DSI of 5 is characteristic of a dormant terminal bud with complete growth cessation **(E)**. DSI indexes of the 35S:*MdDAM1* transgenic lines and the non-transformed (NT) control at 13 (closed bars) 20 (open bars), and 27 weeks (gray bars) after pruning. Significance of the switch to dormancy relative to the NT control was determined using Student’s t-test. ****P value (two-tailed) < 0.005 **(F)**.

**Figure 3:**
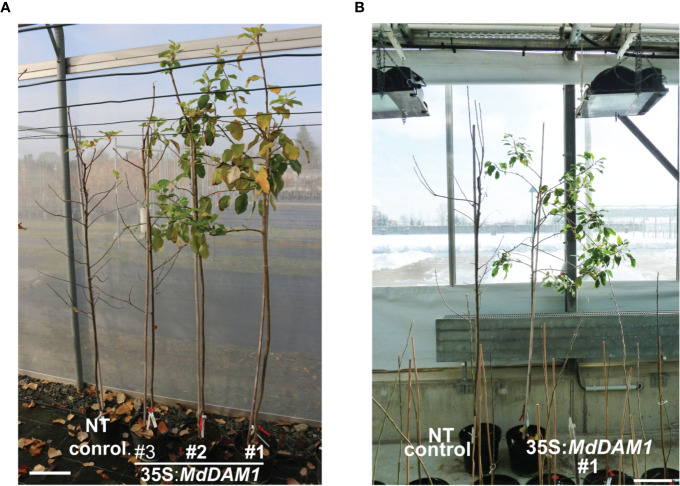
The 35S:*MdDAM1* transgenic lines never enter in dormancy and show an evergrowing/evergreen phenotype even in harsh winter conditions. Picture of the transgenic trees 35S:*MdDAM1* and the NT control taken in autumn at the end of November **(A)** and in winter at the end of February **(B)** seven and six years after transformation, respectively. The trees are grown in GMO tunnels with protective nets **(A)** and inside a GMO glasshouse in **(B)** with heavy snow conditions outside. The bar in **(A, B)** =30 cm.

In order to study the function of *MdDAM1* in dormancy we constitutively expressed by transgenesis the coding sequence from ‘Golden Delicious’ using the CaMV35S promoter. We hypothesized that, either *MdDAM1* gene expression will be overexpressed constitutively in all tissues, or will eventually trigger co-suppression of the endogenous *MdDAM1*, particularly in buds (**Figure 2A**). The construct was introduced in apple using *Agrobacterium*-mediated transformation and four independent lines growing on kanamycin selective medium were selected for further molecular characterization. We confirmed by Southern blot analysis several T-DNA integrations in lines 35S:*MdDAM1*#1, #2, and #4 and a single insertion event in line #3 (**Figure S3**). Although the band pattern in lines 35S:*MdDAM1*#2 and #4 seems similar (**Figure S3C**), it is rather unlikely that these lines are genetically identical since they were selected from different explants. We considered the four selected 35S:*MdDAM1* transgenic trees as independent T-DNA insertion lines. After micrografting to a rootstock, the transgenic lines as well as the non-transformed control plants were transferred to soil and grown in a glasshouse. The transgenic lines were subjected to phenotypic characterization by visualizing the plant growth fitness, the tree architecture, the capacity to enter in dormancy in autumn, the terminal bud formation and the ability of those buds to break in spring and to form flowers. After two years of growth the most striking and evident phenotype that we observed was the incapacity of the transgenic lines 35S:*MdDAM1*#1, #2, and #4 to enter in dormancy in autumn compared to the non-transformed (NT) controls (**Figure 2B**). During the second growing season in the glasshouse all these three 35S:*MdDAM1* transgenic lines produced between 4 and 5 lateral branches (side shoots) whereas the control plants consisted only of a single main shoot except of one plant which developed one single lateral branch too (**Figure 2B**). In the third year the three transgenic lines constantly produced new shoots and flowers regardless of the season whereas the NT control plants and the line 35S:*MdDAM1*#3 entered in dormancy in autumn. Additionally, the buds produced in the lines 35S:*MdDAM1*#1, #2, and #4 never became dormant and continuously opened to let a new branch grow sometimes with a flower cluster formed simultaneously (**Figures 2C, D**). These observations clearly suggested that the lines 35S:*MdDAM1*#1, #2, and #4 lost their ability to enter in dormancy. In order to measure and assess the capacity of these three transgenic lines to switch from a vegetative stage to dormancy measure we assigned an index that we call DSI (dormancy switch index). A low DSI indicates a strong vegetative growth during the cold season, namely, from November to February whereas a high DSI of 4–5 refers to growth cessation (**Figure 2E**). All three transgenic lines 35S:*MdDAM1*#1, #2, and #4 showed a significantly lower DSI (two-tailed P value <0.005) compared to the NT control trees as they stayed at the vegetative stage at the end of autumn and during winter corresponding to the periods 13 and 20 to 27 weeks after pruning, respectively (**Figure 2F**). In contrast, NT control trees and plants of the line 35S:*MdDAM1*#3 had a high DSI close to 5 and entered in dormancy and vegetative growth ceased completely already 13 weeks after pruning. Remarkably, the transgenic lines 35S:*MdDAM1*#1, #2, and #4 stayed green, never ceased to grow, and produced leaves, flowers and new shoots during all successive winters whether grown in a glasshouse with controlled temperature or when transferred in a net house exposed to external frost and negative temperatures (**Figures 3A, B**). The transgenic lines 35S:*MdDAM1*#1, #2, and #4 showed an evergreen or evergrowing phenotype and are deficient in growth cessation due to a loss of dormancy.

### The Loss of Dormancy and Growth Cessation in Lines #1, #2, and #4 Is Due to Co-Suppression of *MdDAM1*

**Figure 4:**
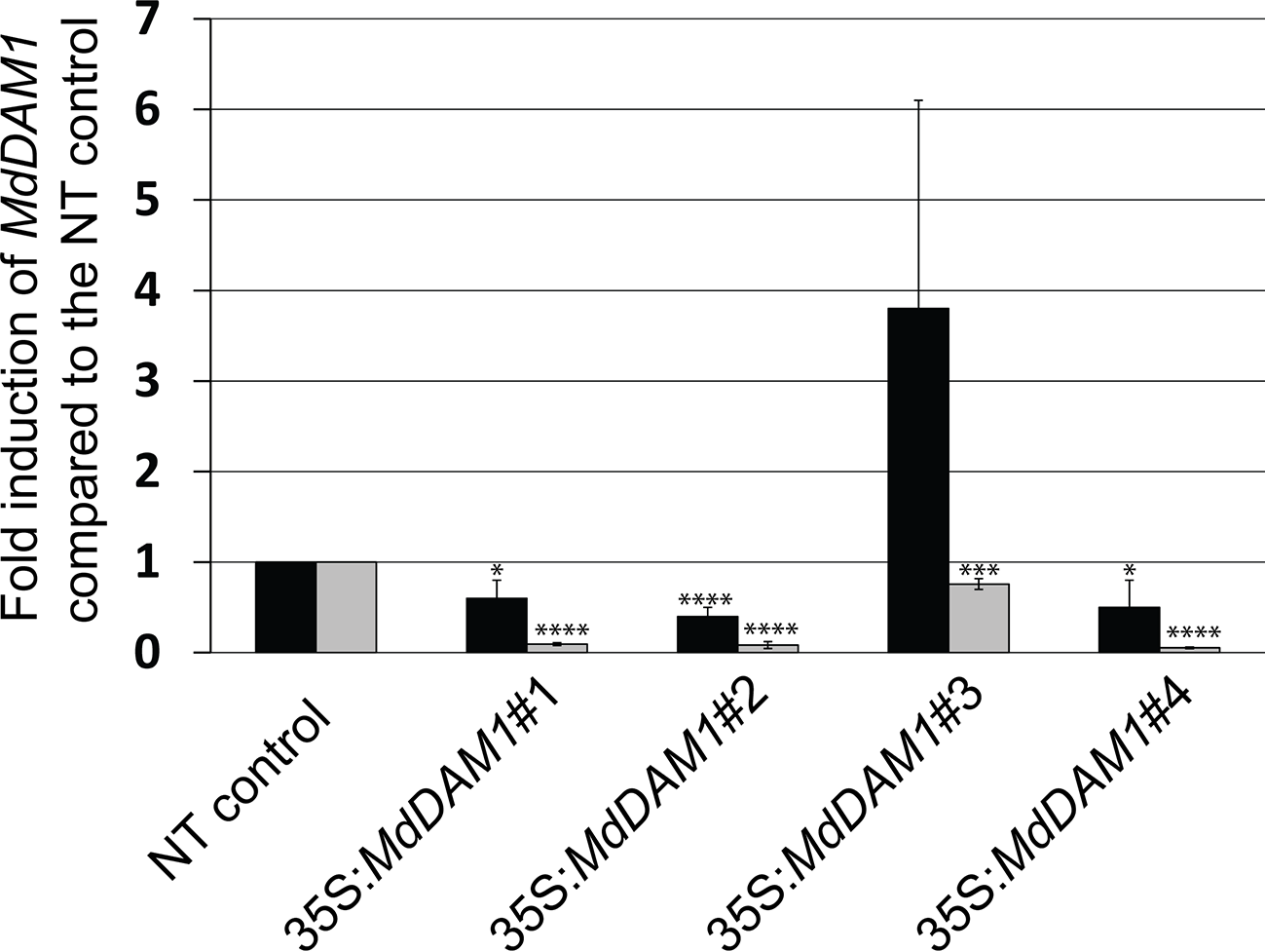
*MdDAM1* gene expression levels in buds of the *MdDAM1* transgenic trees. The relative expression values of *MdDAM1* in terminal buds of transgenic trees 35S:*MdDAM1*#1, #2, #3 and #4 harvested in October (black bars) and February (grey bars) were normalized to *MdEF1* alpha and shown as fold induction compared to the NT control. Error bars represent standard deviation from three biological replicates. Significance levels (Student’s t-test) relative to the NT control expressed as P values (two-tailed): *P < 0.05, ***P < 0.01 and ****P < 0.005.

**Figure 5:**
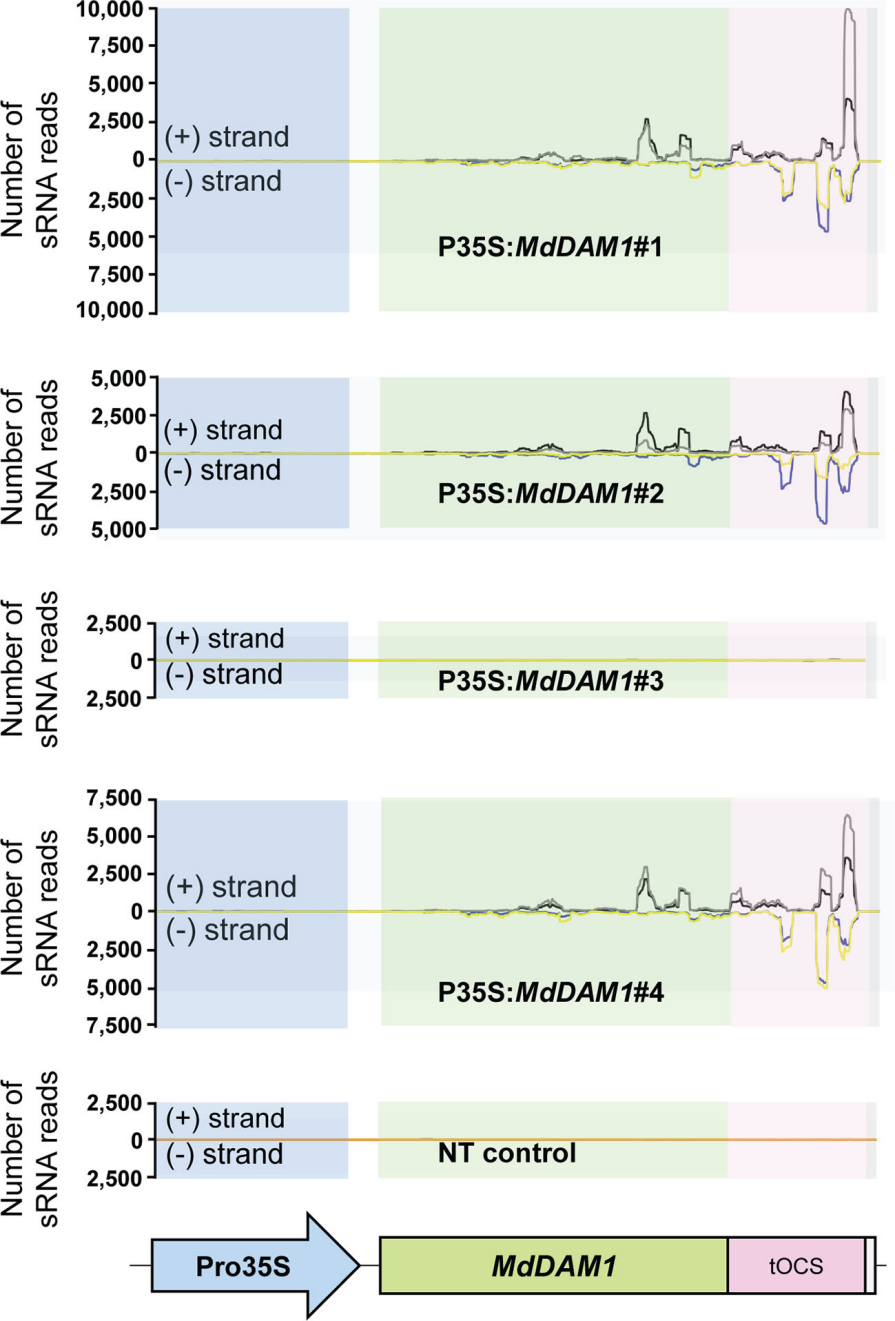
Abundance of small RNAs mapping on the T-DNA inserted in *MdDAM1* transgenic lines. The number of small RNA (sRNA) mapping in sense (+ strand) or in antisense (− strand) of the T-DNA comprising the Promoter (Pro35S), the *MdDAM1* coding region and the octopine synthase terminator (tOCS) are indicated. The colored lines indicate the number of sRNA ranging from 21 to 24 nt length expressed in the buds of the transgenic lines in October (sense in black, antisense in blue) and February (sense in grey, antisense in yellow). The flat lines shown in the NT control and in line P35S:*MdDAM1*#3 indicate that there were no sRNAs mapping to the T-DNA cassette. The sRNA abundance values indicated for each line is the mean from three independent biological replicates.

We checked the *MdDAM1* transcript levels in the transgenic lines 35S:*MdDAM1*#1, #2, #3, and #4 as well as the NT control by qRT-PCR using RNA extracted from terminal buds harvested at the beginning and at the end of the dormancy period corresponding to October and February, respectively. At the beginning of dormancy *MdDAM1* gene expression levels were higher in line 35S:*MdDAM1*#3 compared to the NT control lines. In contrast, the other lines, namely, 35S:*MdDAM1*#1, #2, and #4 showed a significantly (two-tailed P value <0.005) lower expression of *MdDAM1* in October at which time the levels are normally very high (**Figure 4**). As we hypothesized, expressing in a constitutive manner a gene in apple under the strong CaMV35S promoter usually leads to a mix of transformants either overexpressing the gene introduced or silencing the endogenous copy, a phenomenon known as co-suppression. To verify this we sequenced small RNAs (sRNAs) from buds of the transgenic lines and the NT control trees. The lines 35S:*MdDAM1*#1, #2, and #4 with a low expression of *MdDAM1* showed an accumulation of sRNAs of 21 to 24 nt length in sense and anti-sense orientation on *MdDAM1* coding region and the tOCS terminator. The accumulation of sRNAs deriving from *MdDAM1* coding region inserted with the T-DNA likely triggers in *trans* the endogenous mRNA produced in buds resulting in a constitutive silencing of *MdDAM1*. In contrast, the line 35S:*MdDAM1*#3 and the NT control tree did not produce any sRNA mapping to the T-DNA (**Figure 5**). In sum, we obtained one transgenic line (#3) in which *MdDAM1* was overexpressed from the transgene and three lines (#1, #2 and #4) in which the transgene and the endogenous *MdDAM1* were silenced by a co-suppression mechanism.

### The Transgenic Line 35S:*MdDAM1*#1 Is a Genocopy of the ‘Golden Delicious’ Tree at Bud Break

**Figure 6:**
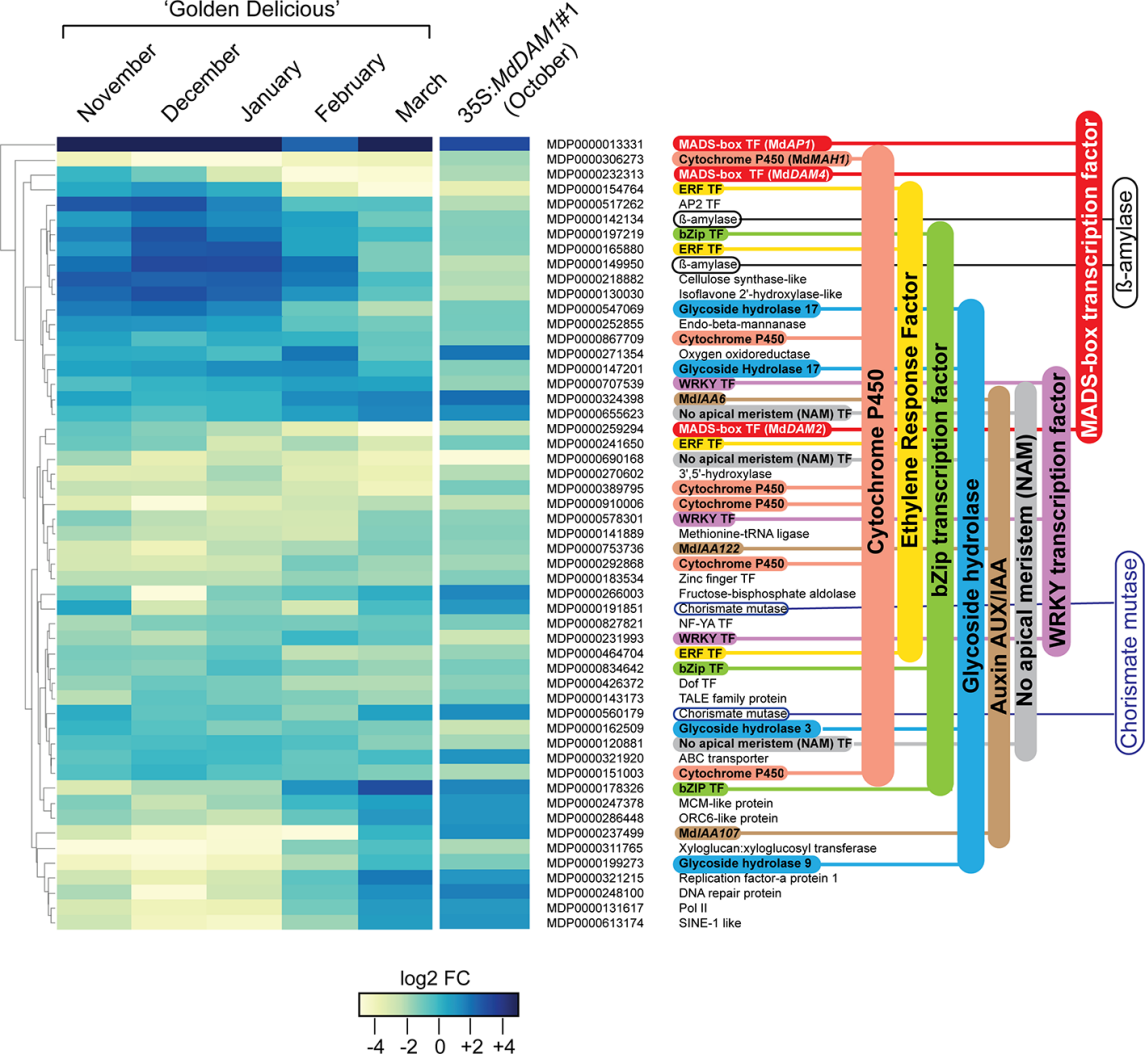
Genes differentially expressed in buds of the transgenic line 35S:*MdDAM1*#1 and their profile in ‘Golden Delicious’ during dormancy. Heatmap indicating 53 genes differentially expressed in buds of the line 35S:*MdDAM1*#1 in autumn (October) compared to the NT control. The fold changes (FC) in gene expression levels of those genes at different time points during bud dormancy of ‘Golden Delicious’ (November to bud break in March) normalized to the October time point were further hierarchically clustered. The values are expressed in log2 FC calculated from a differential RNA-seq data analysis (DESeq2) using three independent biological replicates for each time point. The gene families with more than two members are indicated using a color code.

In order to identify genes that are regulated by *MdDAM1* we performed a differential expression analysis between terminal buds of the representative 35S:*MdDAM1*#1 line and those of the NT control. To avoid a misinterpretation of the data due to the seasonal silencing of *MdDAM1* in March we performed the RNAseq analysis using buds from both genotypes harvested in October. At that period of the year *MdDAM1* expression is normally high in trees growing in normal conditions. A differential gene expression analysis revealed that only 53 genes are differentially expressed in 35S:*MdDAM1*#1 terminal buds compared to those of the NT control (**Figure 6**). Notably, the expression profile of those 53 genes in 35S:*MdDAM1*#1 in October is almost similar to the one of ‘Golden Delicious’ in March at bud break as shown in a RNAseq time course experiment (**Figure 6**). These results suggest that buds from trees in which *MdDAM1* is constitutively silenced behave like those of ‘Golden Delicious’ in March when dormancy is broken. The transgenic line 35S:*MdDAM1*#1 can be considered as a genocopy of ‘Golden Delicious’ at the onset of bud break in spring.

All these 53 differentially expressed genes showed a seasonal gene expression in buds of ‘Golden Delicious’ with the majority overexpressed during cold periods in December and January and gradually repressed in March when spring arrives (**Figure 6**). The majority of the 53 genes belong to gene families, namely, six genes belonging to the cytochrome P450, four Ethylene Responsive Factors (ERF), three bZIP transcription factors, three glycoside hydrolase, three auxin/indole-3-acetic acid (Aux/IAA) genes, three MADS-box transcription factors, three WRKY transcription factors, two ß-amylases, and two chorismate mutases. The twenty remaining genes encode either enzymes with diverse functions or other transcription factors of the AP2, Zinc finger, NF-YA, and Dof family. Our results clearly indicate that genes which should normally be either expressed or repressed at bud break in normal conditions constitutively show a differentially expressed status in the transgenic 35S:*MdDAM1*#1 line and might explain why trees in which *MdDAM1* is silenced never enter in dormancy.

### Both MADS-Box Genes *MdDAM1* and *MdDAM4* Are Involved in Bud Dormancy

**Figure 7:**
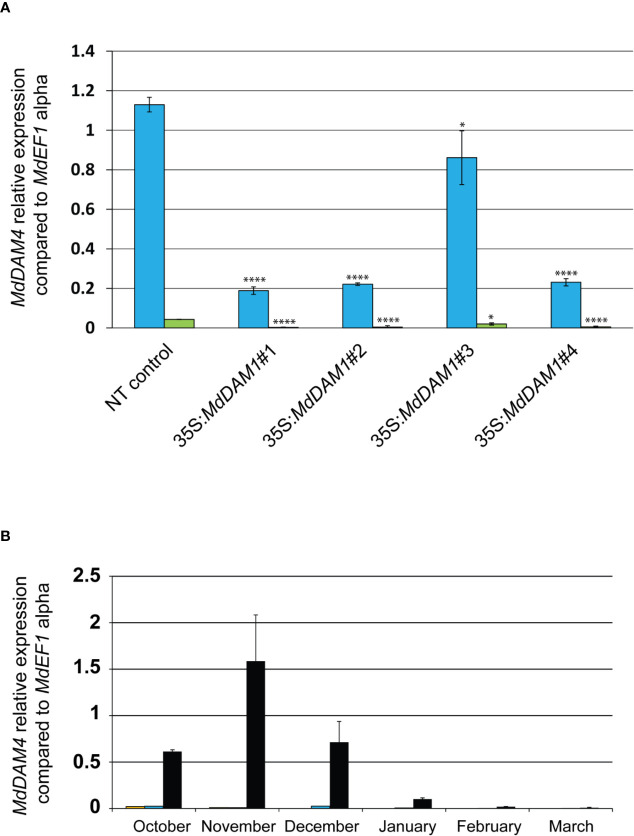
Expression of *MdDAM4* in buds of the *MdDAM1* transgenic lines and during dormancy. The relative expression values of *MdDAM4* in buds of the different transgenic lines *MdDAM1* lines and the NT control in October (blue bars) and February (green bars) are indicated. Significance levels (Student’s t-test) relative to the NT control expressed as P values (two-tailed) were as follows: *P < 0.05 and ****P < 0.005 **(A)**. The expression of *MdDAM4* in buds of ‘Dorsett Golden’ (orange bars), ‘Anna’ (blue bars) and ‘Golden Delicious’ (black bars) cultivars collected at different time points from the onset of dormancy in October to bud break in March is indicated in **(B)**. *MdDAM4* relative expression compared to the housekeeping gene *MdEF1* alpha was determined by qRT-PCR. Error bars represent standard deviation from three biological replicates.

One of the top three most differentially expressed genes (p-value adj. = 6.75^−15^, **Table S2**) during apple bud dormancy is *MdDAM4* (MDP0000232313 and NCBI accession number KT003615), another dormancy-associated MADS-box gene. Interestingly this gene seems to be directly regulated by *MdDAM1* since it is differentially expressed in the line 35S:*MdDAM1*#1. *MdDAM4* is constitutively low when *MdDAM1* is silenced suggesting that *MdDAM1* influences the expression of *MdDAM4*. It is plausible that this regulation is done through transcriptional activity of *MdDAM1* on the promoter of *MdDAM4*. However, due to the high sequence similarity at the nucleotide level between *MdDAM1* and *MdDAM4* we do not exclude that this type of regulation is specific to the transgenic lines 35S:*MdDAM1*#1, 2, and 4 and triggered by the sRNAs deriving from *MdDAM1* locus and targeting *MdDAM4* in a sequence specific manner (**Figure S4**). Nevertheless, when both MADS-box genes are downregulated, transgenic apple trees display an evergreen phenotype as observed in the lines 35S:*MdDAM1*#1, 2, and 4 but not in those when *MdDAM1* is overexpressed in line 35S:*MdDAM1*#3. We checked using qRT-PCR the expression of *MdDAM4* in October and February and found that *MdDAM4* is significantly (two-tailed P value <0.005) lower in lines 35S:*MdDAM1*#1, 2, and 4 compared to the NT control trees already in autumn when buds normally enter in dormancy and high only in the line 35S:*MdDAM1*#3 overexpressing *MdDAM1* (**Figure 7A**). Interestingly, *MdDAM4* expression showed a profile similar to *MdDAM1* in ‘Golden Delicious’ during the course of dormancy in autumn and winter. However, *MdDAM4* is constitutively silenced in the low chill cultivars ‘Dorsett Golden’ and ‘Anna’ suggesting that it is directly correlated to the regulation of the duration of bud dormancy and the onset of bud break in apple (**Figure 7B**). In sum and taken together, these results show that *MdDAM1* is a positive regulator of *MdDAM4* and both genes promote bud dormancy and growth cessation in apple. Therefore, their transcriptional repression favors growth and triggers bud break.

## Discussion

Our results clearly indicate that silencing of *MdDAM1* in apple trees leads to a loss of bud dormancy hence an abolishment of growth cessation that usually occurs in autumn. We demonstrated that, out of four transgenic trees selected for this work, three showed a silencing of *MdDAM1* by co-suppression of the endogenous gene (Napoli et al., 1990; Van Der Krol et al., 1990). The silencing of the endogenous *MdDAM1* gene triggered the formation of sRNAs that are processed mainly from the 3’ terminal part of the inserted T-DNA cassette and which includes the *MdDAM1* coding region itself and the tOCS terminator. The formation of sRNAs in these regions in sense and antisense is a hallmark of post-transcriptional gene silencing occurring in plants during transgenesis (De Paoli et al., 2009) therefore validating the silencing of *MdDAM1* in the transgenic lines #1, #2 and #4 by RNA interference (RNAi). This RNA silencing could be either caused by the threshold of *MdDAM1* transcripts generated by the use of a combination of the strong CaMV35S promoter and the tOCS terminator as described in other transgenic apple studies (Yao et al., 2018), or due to the numerous T-DNA insertions that occurred during transgenesis in lines #1, #2 and #4 (Schubert et al., 2004). In contrast, line #3 showed a single T-DNA insertion (**Figure S3**) and a higher expression of *MdDAM1* compared to the NT control and did not reach a sufficient threshold to trigger RNA silencing of the endogenous copy. Furthermore, no sRNAs were detected in line 35S:*MdDAM1*#3. In conclusion, we succeeded in obtaining both silenced lines and an overexpressor of *MdDAM1* by using a single T-DNA construct expressing *MdDAM1* in sense orientation. This experimental set up allowed us to compare the phenotype of the transgenic trees based solely on the level of *MdDAM1* expression in buds.

The transgenic lines 35S:*MdDAM1*#1, #2 and #4 constantly produced green leaves regardless of the season and never ceased to grow even during the winter period. Furthermore, the buds of these three lines never became dormant and constantly broke to produce new shoots sometimes with a flower cluster emerging even in winter. Interestingly, this phenotype is reminiscent to the one of the *evergrowing* (*evg*) peach mutant (previously known as *evergreen*) that is insensitive to cessation of growth under short days and low temperatures (Rodriguez-A. et al., 1994). The peach *evg* tree mutant has a deletion of a cluster of six MADS-box genes and collinearity between *MdDAM1* as well as *MdDAM4* genomic regions and the *EVG* locus was demonstrated (Porto et al., 2016) suggesting a conserved role in bud dormancy in *Prunus* species. The correlation between the silencing of *MdDAM1* and the concomitant downregulation *MdDAM4* in our transgenic trees resulting in a loss of dormancy remarkably resembles the peach *evg* mutant situation. We therefore suggest that silencing of *MdDAM1* and *MdDAM4* negatively regulate bud dormancy by inhibiting growth cessation and their role is evolutionary conserved among *Prunus* species. Furthermore, in contrast to the peach *evg* mutant, we provide evidence that silencing only these two DAM genes out of the six identified in apple is sufficient to obtain an evergrowing phenotype. However, it is still unclear how the different *DAM* proteins interact in peach and apple. One possibility is that, as a transcription factor, *MdDAM1* directly regulate *MdDAM4*. Two CArG MADS-box cis-element motifs were found on the promoter region of *MdDAM4* and could serve as a binding site for *MdDAM1* as well as other MADS-box genes such as *MdDAM2* also expressed in apple buds and differentially expressed in the 35S:*MdDAM1* transgenic line (Porto et al., 2016). Future works will show if the role of *MdDAM1* in dormancy is a conserved mechanism in other *Rosaceae* species although the variety of bud morphology (vegetative/generative versus mixed) in fruit trees belonging to this family can make the functional and comparative studies challenging.

We provided evidence that *MdDAM1* expression in the low-chill cultivars ‘Anna’ and ‘Dorsett Golden’ is drastically reduced in January and totally silenced in February one month earlier than in ‘Golden Delicious’. There is therefore a strong correlation between *MdDAM1* expression and bud break. This difference in *MdDAM1* gene expression could be due to a mutation of a single nucleotide in the cultivars ‘Anna’ and ‘Dorsett Golden’ leading to the substitution of a methionine (M) to valine (V) at position 194 (**Figure S2**). However, this single nucleotide polymorphism (SNP) is outside of the conserved MADS and K-box domains and there is no report indicating the potential effects of such mutation in plant MADS-box genes. Additional work is required to ascertain if the decrease of *MdDAM1* gene expression in low-chill apple cultivars is due to this mutation or other effects such as epigenetic gene regulation at the transcriptional level as shown for the *DAM6* gene present on the *EVG* locus (Leida et al., 2012). It is also plausible that hormones such as abscisic acid (ABA) play a role in the regulation of *MdDAM1* as shown for *Maleae* and *Prunus* DAM genes, namely, *PpDAM1/PpDAM3* in Japanese pear (*P. prifolia*) and *PmDAM6* in Japanese apricot (*Prunus mume*) (Tuan et al., 2017; Falavigna et al., 2019; Yamane et al., 2019; Yang et al., 2020). ABA-mediated regulation of MADS-box genes orthologs of the *SVP* clade is also important in the distant tree hybrid aspen to promote growth cessation and bud dormancy (Singh et al., 2019). The measurement of ABA content in buds of ‘Anna’ and ‘Dorsett Golden’ would reveal if this mechanism is also important for the low expression of *MdDAM1* in low-chill cultivars and would provide, besides epigenetics, an explanation to the results obtained in our study. Whether or not regulated at the transcriptional and/or at the post-transcriptional level *MdDAM1* could be certainly used as a marker in breeding programs that aims at producing new low-chill apple cultivars. We expect that the need for elite apple varieties such as ‘Golden Delicious’ with lower chilling requirements will increase with the milder winters foreseen in the next decades as a direct consequence of climate change. Early genetic studies using crosses ‘Anna’ × ‘Golden Delicious’ have already provided evidence that low chilling requirements in ‘Anna’ is controlled by at least one major gene modulated by the interaction of other minor genes (Hauagge and Cummins, 1991). More recently, a QTL analysis identified *MdDAM1* as a strong candidate gene controlling chilling perception in apple (Allard et al., 2016). Alternatively, new plant breeding techniques such as genome editing of *MdDAM1* gene could speed up the production of low chilling cultivars variants in elite cultivars (Zhang et al., 2019).

Silencing of only *MdDAM1* resulted in a dramatic phenotype in apple. This could be due to the key role of MADS-box transcription factors in the regulation of complex gene regulatory network in plants (Castelán-Muñoz et al., 2019). Transcriptional profiling of the 35S:*MdDAM1* transgenic line showed differential expression of 53 genes that could be part of this regulatory network. Interestingly, the majority of these mRNA transcripts belong to few genes families suggesting a quite restricted number of genes regulated by *MdDAM1*, either positively or negatively. Some gene families, such as the Cytochrome P450 family, identified in this work are large and involved in numerous plant development and defense mechanisms (Xu et al., 2015). The role of Cytochrome P450 in bud dormancy and cessation of growth is unknown. Among the six Cytochrome P450 genes differentially expressed in the 35S:*MdDAM1*#1 transgenic line only *MdAH1* was previously described. This gene is potentially involved in the oxidation of alkane to secondary alcohol and ketone in the cuticular wax pathway (Li et al., 2019). Transcription factors belonging to the WRKY gene family, with known roles in biotic and abiotic stresses, were also differentially expressed (Phukan et al., 2016). Although their role in bud dormancy in peach was recently hypothesized (Chen et al., 2016) a more rational explanation to their seasonal expression profile is their regulation by cold stress as described in grapevine (Wang et al., 2014). Other transcription factor gene families were also differentially expressed in the 35S:*MdDAM1*#1 transgenic tree. Four Ethylene response factors (ERF) genes that expanded considerably in apple were identified (Girardi et al., 2013). Among them three were identified as significantly upregulated under various abiotic-stress treatments (Zhao et al., 2012). The fourth ERF gene MDP0000464704 was shown to be regulated during apple flower bud formation in response to shoot-bending conditions (Xing et al., 2019). Additionally, two transcription factor families, namely the no apical meristem (NAM) and the bZIP, have three gene members each that are differentially expressed. The NAM gene MDP0000690168 is upregulated in buds of so-called ‘ON’ trees in which floral induction is inhibited due to biennial bearing whereas it is constitutively downregulated in the 35S:*MdDAM1*#1 and silenced in spring in ‘Golden Delicious’ (Guitton et al., 2016). This is additional evidence that the regulatory network controlled by *MdDAM1* is involved in processes related to bud break. Finally, silencing of *MdDAM1* has an effect on the auxin pathway since the genes *MdIAA107* (MDP0000237499), *MdIAA122* (MDP0000753736), and *MdIAA6* (MDP0000324398) are all upregulated in the 35S:*MdDAM1*#1 as well as in spring (March) in broken ‘Golden Delicious’ buds.

In conclusion, *MdDAM1* is a crucial regulator of growth cessation and bud break in apple by controlling the expression of certain gene families. A better understanding of *MdDAM1* regulatory network could help to open new routes for manipulating bud dormancy in fruit trees. Finally, it will be of importance to generate trees in which *MdDAM1* and *MdDAM4* are silenced both individually and simultaneously using genome editing techniques to assess their synergetic effect on growth cessation and bud dormancy.

## Data Availability Statement

The original contributions presented in the study are publicly available at NCBI under the project accession PRJNA374502. This data can be found here: https://www.ncbi.nlm.nih.gov/bioproject/?term=PRJNA374502.

## Author Contributions

MM, EA, GM, KW, M-VH, HF, and AS-A contributed to the design of the research. EA, GM, and KW performed the experiments. MM, EA, HF, and AS-A analyzed the data. AS-A and HF wrote the manuscript. All authors contributed to the article and approved the submitted version.

## Funding

This work was supported by the Autonomous Province of Trento (“TranscrApple”, grandi progetti 2012 to AS-A).

## Conflict of Interest

The authors declare that the research was conducted in the absence of any commercial or financial relationships that could be construed as a potential conflict of interest.
