# Supplementary material for: The MADS-Box Gene MdDAM1 Controls Growth Cessation and Bud Dormancy in Apple
Source: Front Plant Sci. 2020 Jul 7;11:1003. doi: 10.3389/fpls.2020.01003 (PMC7358357; doi:10.3389/fpls.2020.01003)
Supplement: Supplementary file 1 [file DataSheet_1.pdf]

Supplementary Material

Table S1. Primer sequences.

Primer	Sequence 5'-3'	Purpose
***MdDAM1*-Fq**	TCAAGCGTGGGTACGTTGCTTC	qRT-PCR
***MdDAM1*-Rq**	GATGACCTGAGCGATAAAGTTGGC	qRT-PCR and genotyping of transgenic plants
***MdDAM4*-Fq**	CATACTGGTGGGGAAAAATCG	qRT-PCR
***MdDAM4*-Rq**	CTCAGCTTGCGGGTCTTATC	qRT-PCR
**MdEF1alpha_F**	TCAAGCGTGGGTACGTTGCTTC	qRT-PCR
**MdEF1alpha_R**	GATGACCTGAGCGATAAAGTTGGC	qRT-PCR
**nptII_F**	ACAAGATGGATTGCACGCAGG	Genotyping of transgenic trees
**nptII_R**	AACTCGTCAAGAAGGCGATAG	Genotyping of transgenic trees
**tOCS_R**	ATCATGCGATCTAGGCGTC	Genotyping of transgenic trees
**virG_F**	GCCGGGGCGAGACCATAG	Genotyping of transgenic trees
**virG_R**	CGCACGCGCAAGGCAACC	Genotyping of transgenic trees

Table S2. Fold Changes of the 53 differentially expressed genes in line P35S:MdDAM1#1 and their corresponding level of expression in 'Golden delicious' during a bud dormancy time course

			log2FC in 'Golden delicious' normalized to "October" values											
Gene ID	log2FC in P35S:*MdDAM1*#1 compared to NT control	D=Down-regulated; U=Up-regulated	November	December	January	February	March	Gene description	Base Mean	log2Fold Change	lfcSE	stat	p-value	p-adjusted value
MDP0000013331	3.102008678	U	5.323777361	5.219265464	5.132224509	2.382071306	5.122451642	MADS-box TF (MdAP1)	37.93058	3.102009	0.422284	7.345787	2.05E-13	1.20936E-10
MDP0000306273	-1.765324523	D	-4.039082471	-8.411557551	-5.081813402	-4.077032721	-3.914831834	Cytochrome P450	127.8228	-1.76532	0.435069	-4.05758	4.96E-05	0.002414223
MDP0000232313	-2.028923988	D	0.007427254	-0.991325717	-3.104317329	-7.041121229	-4.74071452	MADS-box TF (MdDAM4)	102.4041	-2.02892	0.234514	-8.65163	5.08E-18	6.75369E-15
MDP0000154764	-3.574346057	D	0.442054783	1.07493231	0.283823052	-3.628397812	-5.866732565	ERF TF	76.98169	-3.57435	0.423111	-8.44777	2.97E-17	3.35142E-14
MDP0000517262	-2.174799841	D	2.725017536	2.89907733	1.590991199	-0.477453471	-0.450648799	AP2 TF	107.3594	-1.09719	0.278619	-3.93794	8.22E-05	0.003514481
MDP0000142134	-1.371959521	D	0.885393104	1.878934016	1.387006718	0.730400012	-0.945293083	Beta-amylase	418.1697	-1.37196	0.166457	-8.24214	1.69E-16	1.70286E-13
MDP0000197219	-1.320494194	D	1.646206286	2.865225196	1.896792402	0.554264209	-0.118793867	bZip TF	243.4806	-1.32049	0.249784	-5.28655	1.25E-07	1.77202E-05
MDP0000165880	-1.359287916	D	1.090650763	2.645518221	2.745179792	0.581787466	-1.192573437	ERF TF	181.0693	-1.35929	0.201182	-6.75651	1.41E-11	5.48431E-09
MDP0000149950	-2.329176978	D	2.035986436	3.152300132	3.080024204	2.036100625	-1.194778901	Beta-amylase	171.1249	-2.56044	0.279437	-9.16287	5.05E-20	8.55607E-17
MDP0000218882	-2.076243815	D	2.608407537	2.408170486	2.486722227	1.873075336	-0.256698457	Cellulose synthase-like	106.0868	-2.07624	0.498984	-4.16094	3.17E-05	0.00167449
MDP0000130030	-2.305458588	D	2.231309487	2.916064311	2.362873672	1.159869709	-0.656154546	Isoflavone 2'-hydroxylase-like	35.18403	-2.30546	0.467564	-4.93079	8.19E-07	8.84208E-05
MDP0000547069	-1.055181541	D	1.777410171	2.057836188	1.49467537	-0.858322594	-2.193400201	Glycoside hydrolase 17	314.2396	-1.05518	0.209225	-5.04329	4.58E-07	5.30961E-05
MDP0000252855	-1.043430114	D	1.13305786	1.072721136	0.736229376	-0.637396175	-1.037033923	Endo-beta-mannanase	216.0798	-1.04343	0.289367	-3.6059	0.000311	0.009264854
MDP0000867709	-1.247196782	D	0.694345511	-0.322923738	0.291576483	0.185218238	-0.776991646	Cytochrome P450	434.9515	-1.2472	0.155632	-8.01377	1.11E-15	9.8655E-13
MDP0000271354	1.936128804	U	0.347576809	0.303761663	-0.265744914	1.895238107	-0.89192327	Oxygen oxidoreductase	68.95418	1.936129	0.39853	4.858182	1.18E-06	0.000117669
MDP0000147201	-1.345055444	D	0.587634219	0.811897925	1.338263964	1.404522944	-0.051528816	Glycoside Hydrolase 17	44.9735	-1.32307	0.35982	-3.67702	0.000236	0.007590104
MDP0000707539	-1.628383451	D	-0.338049421	0.148465037	0.367685763	0.770688765	0.658012873	WRKY TF	44.64627	-1.62838	0.448463	-3.63103	0.000282	0.008634265
MDP0000324398	2.127427854	U	0.616135408	0.034562742	0.048585632	1.398324732	1.614345852	MdIAA6	37.33274	2.127428	0.444985	4.780899	1.75E-06	0.00016373
MDP0000655623	1.335121018	U	-0.094125008	-0.577521039	0.061809461	0.762454064	1.492695989	No apical meristem (NAM) TF	41.93428	1.335121	0.368629	3.621857	0.000292	0.008900814
MDP0000259294	-2.422074935	D	-0.724518138	-1.334792067	-2.172188419	-3.466670797	-4.941138902	MADS-box TF (MdDAM2)	112.6455	-2.42207	0.234916	-10.3104	6.33E-25	2.35599E-21
MDP0000241650	-1.052397594	D	-0.841600326	-1.226951883	-3.044196769	-1.923599425	-3.115033118	ERF TF	197.6707	-1.0524	0.19117	-5.50503	3.69E-08	5.82943E-06
MDP0000690168	-4.903849779	D	-1.807653632	-3.574704992	-2.541159049	-3.165751151	-4.018894702	No apical meristem (NAM) TF	110.5939	-4.90385	1.137845	-4.30977	1.63E-05	0.001019609
MDP0000270602	-2.156593915	D	-3.388673248	-3.283569314	-1.875339737	-3.324889771	-3.750827026	3',5'-hydroxylase	101.5539	-2.15659	0.346884	-6.21704	5.07E-10	1.33832E-07
MDP0000389795	-1.131347002	D	-2.443717436	-2.582504459	-2.242040503	-2.931616208	-4.003158918	Cytochrome P450	888.8723	-1.13135	0.199803	-5.66233	1.49E-08	2.67418E-06
MDP0000910006	-2.34829143	D	-3.34883594	-4.93302134	-3.34693843	-2.719046885	-2.226485811	Cytochrome P450	57.51566	-2.34829	0.384325	-6.11017	9.95E-10	2.43878E-07
MDP0000578301	-1.459017174	D	-1.310229829	-2.280640454	-2.851255867	-2.891971868	-1.402629005	WRKY Family Protein	88.48401	-1.45902	0.329133	-4.43292	9.3E-06	0.000652115
MDP0000141889	-1.380175295	D	-1.947680916	-2.408882289	-2.630593672	-3.002086054	-1.289968716	Methionine-tRNA ligase	61.92002	-1.38018	0.379629	-3.63559	0.000277	0.008530013
MDP0000753736	-1.512239083	D	-3.007757943	-3.943915646	-2.279345682	-1.938831751	-1.201866181	MdIAA122	65.38263	-1.51224	0.342544	-4.41473	1.01E-05	0.000691185
MDP0000292868	-1.811121443	D	-2.953326503	-3.110207197	-2.726705207	-1.108773467	-1.78001353	Cytochrome P450	44.07092	-1.57627	0.387996	-4.06258	4.85E-05	0.002378614
MDP0000183534	-1.302731934	D	-2.243759948	-2.249209535	-2.342569496	-1.579905161	-1.897664167	Zinc finger TF	99.92978	-1.30273	0.352931	-3.69118	0.000223	0.007293178
MDP0000266003	1.50319907	U	-0.844407507	-5.408951138	-1.231631753	-0.656397599	0.283498996	Fructose-bisphosphate aldolase	47.49853	1.503199	0.411233	3.65535	0.000257	0.008106958
MDP0000191851	1.008989601	U	0.572189116	-2.865071024	-1.505574819	-2.954217557	-0.207871749	Chorismate mutase	88.67534	1.00899	0.275978	3.656056	0.000256	0.008091534
MDP0000827821	-1.180624206	D	-1.931172766	-1.260113384	-1.017789606	-1.1115674	-0.851993582	NF-YA TF	132.8057	-1.18062	0.27845	-4.23999	2.24E-05	0.001284873
MDP0000231993	-2.665595949	D	-1.717597147	-2.295361437	-1.244964068	-0.004026756	-0.723762313	WRKY TF	58.11666	-2.6656	0.498378	-5.34854	8.87E-08	1.30021E-05
MDP0000464704	-1.646477394	D	-1.157628935	-1.247657156	-0.596736464	-2.373154482	-2.088648847	ERF TF	72.46021	-1.64648	0.332563	-4.95088	7.39E-07	8.16553E-05
MDP0000834642	-1.165073996	D	-1.214282665	-1.414913186	-0.281169537	-1.167032022	-1.702733755	bZip TF	103.3053	-1.16507	0.304648	-3.82432	0.000131	0.005004377
MDP0000426372	-1.414807605	D	-1.633041024	-0.864100318	-1.139067074	-2.108664718	-2.144396104	Dof TF	189.3233	-1.41481	0.212346	-6.66276	2.69E-11	9.62466E-09
MDP0000143173	-1.154786705	D	-2.235910208	-0.595539268	-1.101857781	-1.27128824	-1.828455658	TALE family protein	506.8575	-1.15479	0.307094	-3.76037	0.00017	0.006043064
MDP0000560179	1.349486767	U	0.349763136	-0.684554066	-0.44055133	-1.579331271	0.669154972	Chorismate mutase	101.7293	1.349487	0.247617	5.449891	5.04E-08	7.72543E-06
MDP0000162509	-2.567049596	D	0.064475533	-0.531875798	-1.297254999	-0.756306464	-1.199042086	Glycoside hydrolase 3	64.17803	-2.56705	0.422768	-6.072	1.26E-09	3.01624E-07
MDP0000120881	-1.898132971	D	-0.375173922	-0.245653905	-0.093083013	-0.354164847	-1.425216763	No apical meristem (NAM) TF	83.05418	-1.89813	0.500578	-3.79188	0.00015	0.005530176
MDP0000321920	1.171056965	U	-0.659820129	-0.055235414	-0.140245638	-0.782139467	-0.175959053	ABC transporter	137.251	1.171057	0.29213	4.008689	6.11E-05	0.00282507
MDP0000151003	-1.989454396	D	-0.414526276	0.143390658	-0.5196733	-0.778776715	-1.267013019	Cytochrome p450	164.4592	-1.98945	0.336236	-5.91683	3.28E-09	6.98539E-07
MDP0000178326	1.534535391	U	-3.140760595	-1.932351608	-1.920647137	1.158471155	3.03137429	bZIP TF	46.36076	1.534535	0.387279	3.962349	7.42E-05	0.003257134
MDP0000247378	1.098243896	U	-1.286517248	-2.489701998	-1.737108963	-0.058776161	0.713396245	MCM-like protein	82.623	1.098244	0.30428	3.609314	0.000307	0.009199606
MDP0000286448	1.124304427	U	-1.492120471	-2.112714277	-2.515688929	-0.770997792	1.028942584	ORC6-like protein	86.75472	1.124304	0.304471	3.692644	0.000222	0.007267316
MDP0000237499	1.256600187	U	-2.894779743	-4.331110302	-4.725614272	-5.704418021	0.071164547	MdIAA107	109.5031	1.2566	0.31972	3.93031	8.48E-05	0.003603083
MDP0000311765	-2.0108648	D	-6.10911244	-6.857269148	-4.671908917	-1.346727795	-0.361391551	Xyloglucan:xyloglucosyl transferase	114.3166	-2.01086	0.413523	-4.86276	1.16E-06	0.000115596
MDP0000199273	-1.204240417	D	-4.161101355	-4.716180565	-4.134476381	-2.080775132	-0.089402194	Glycoside hydrolase 9	298.7596	-1.20424	0.193216	-6.23261	4.59E-10	1.22047E-07
MDP0000321215	1.072584438	U	-2.75037237	-3.678874159	-2.912148022	-0.597493537	1.86017383	Replication factor-a protein 1	188.0246	1.072584	0.242664	4.420047	9.87E-06	0.000680744
MDP0000248100	1.713224259	U	-2.005826279	-4.815899039	-2.746572609	-0.777840906	1.256983429	DNA repair protein	35.17381	1.713224	0.468556	3.656391	0.000256	0.008088056
MDP0000131617	1.013233752	U	-3.04172421	-3.956660686	-3.805042828	-1.047892226	0.944045674	Pol II	183.4405	1.013234	0.251191	4.033715	5.49E-05	0.002614998
MDP0000613174	1.130680656	U	-2.665347219	-4.138882759	-4.399481132	-1.720746076	0.815811048	SINE-1 like	355.2206	1.130681	0.283825	3.983728	6.78E-05	0.003045075

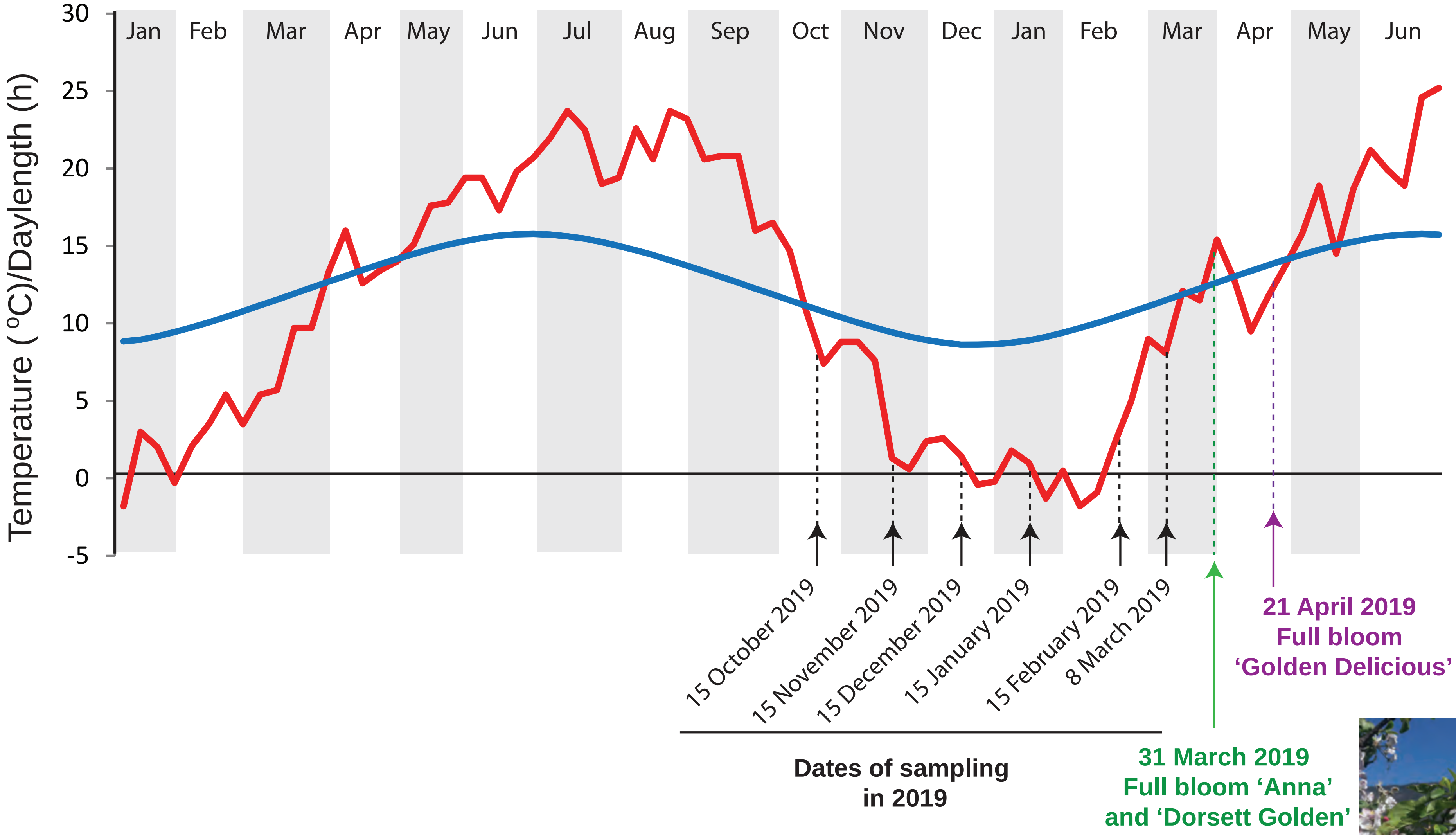

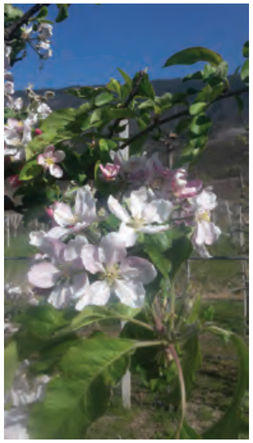

Figure S1. Environmental parameters during the apple bud dormancy time course experiment.

Temperatures in °C and day lengths in hours (h), indicated by a red and a blue line, respectively, were recorded each month from 2011 to 2019 at the orchard and the mean value plotted. Months are indicated by abbreviations. Buds from the cultivars 'Golden Delicious', 'Anna', and 'Dorsett Golden' were harvested at six time points from October to March as indicated by vertical arrows. The date of full bloom in 2019 is also indicated for each cultivar.

A

```
MdDAM1_’Golden_delicious’     ATGAAGATCAAGATAAAAAAGATCGACTACTTGCCGGCAAGGCAGGTGAC
MdDAM1_’Dorsett_Golden’       ATGAAGATCAAGATAAAAAAGATCGACTACTTGCCGGCAAGGCAGGTGAC
MdDAM1-‘Anna’                 ATGAAGATCAAGATAAAAAAGATCGACTACTTGCCGGCAAGGCAGGTGAC
                              **************************************************

MdDAM1_’Golden_delicious’     CTTCTCAAAGAGGAGAAGGGGGATTTTCAAGAAAGCTGGAGAGCTGTCGA
MdDAM1_’Dorsett_Golden’       CTTCTCAAAGAGGAGAAGGGGGATTTTCAAGAAAGCTGGAGAGCTGTCGA
MdDAM1-‘Anna’                 CTTCTCAAAGAGGAGAAGGGGGATTTTCAAGAAAGCTGGAGAGCTGTCGA
                              **************************************************

MdDAM1_’Golden_delicious’     TTCTGTGTGAATCTGAAGTTGCTGTTATCATCTTTTCTCAAACTGGCAAG
MdDAM1_’Dorsett_Golden’       TTCTGTGTGAATCTGAAGTTGCTGTTATCATCTTTTCTCAAACTGGCAAG
MdDAM1-‘Anna’                 TTCTGTGTGAATCTGAAGTTGCTGTTATCATCTTTTCTCAAACTGGCAAG
                              **************************************************

MdDAM1_’Golden_delicious’     CTCTTTGATTTCTCAAGCTCCAGCACCAAGGATGTGATTGCAAGGTACAA
MdDAM1_’Dorsett_Golden’       CTCTTTGATTTCTCAAGCTCCAGCACCAAGGATGTGATTGCAAGGTACAA
MdDAM1-‘Anna’                 CTCTTTGATTTCTCAAGCTCCAGCACCAAGGATGTGATTGCAAGGTACAA
                              **************************************************

MdDAM1_’Golden_delicious’     TTCACATATCGGTGGGGAAAAATCGGATCAACCCACGATTCATCAGCTAC
MdDAM1_’Dorsett_Golden’       TTCACATATCGGTGGGGAGAAATCGGATCAACCCACGATTCATCAGCTAC
MdDAM1-‘Anna’                 TTCACATATCGGTGGGGAGAAATCGGATCAACCCACGATTCATCAGCTAC
                              ******************.*******************************

MdDAM1_’Golden_delicious’     AGTTGGAGAAAGAAAACAATATCAGGCTGAGGAAGGAACTTGAGGATAAG
MdDAM1_’Dorsett_Golden’       AGTTGGAGAAAGAAAACAATATCAGGCTGAGGAAGGAACTTGAGGATAAG
MdDAM1-‘Anna’                 AGTTGGAGAAAGAAAACAATATCAGGCTGAGGAAGGAACTTGAGGATAAG
                              **************************************************

MdDAM1_’Golden_delicious’     AGTTGCAAGTTGAGGCAGATGAAGGGTGTGGACCTTGAAGACTTGGATCT
MdDAM1_’Dorsett_Golden’       AGTTGCAAGTTGAGGCAGATGAAGGGTGTGGACCTTGAAGACTTGGATCT
MdDAM1-‘Anna’                 AGTTGCAAGTTGAGGCAGATGAAGGGTGTGGACCTTGAAGACTTGGATCT
                              **************************************************

MdDAM1_’Golden_delicious’     GGATGAACTACAGAAGTTAGAAAAATTGGTGGAAGCAAGCCTTGGCCGTG
MdDAM1_’Dorsett_Golden’       GGATGAACTACAGAAGTTAGAAAAATTGGTGGAAGCAAGCCTTGGCCGTG
MdDAM1-‘Anna’                 GGATGAACTACAGAAGTTAGAAAAATTGGTGGAAGCAAGCCTTGGCCGTG
                              **************************************************

MdDAM1_’Golden_delicious’     TGATTCAAACTAAGGAAGAAAAGATTATGAGTGAGGTTATGGCACTTGAG
MdDAM1_’Dorsett_Golden’       TGATTCAAACTAAGGAAGAAAAGATTATGAGTGAGGTTATGGCACTTGAG
MdDAM1-‘Anna’                 TGATTCAAACTAAGGAAGAAAAGATTATGAGTGAGGTTATGGCACTTGAG
                              **************************************************

MdDAM1_’Golden_delicious’     AAAAAGGGAGCTGAGCTGATAGAAGCTAACAACCAGCTAAGCCACAGGAT
MdDAM1_’Dorsett_Golden’       AAAAAGGGAGCTGAGCTGATAGAAGCTAACAACCAGCTAAGCCACAGGAT
MdDAM1-‘Anna’                 AAAAAGGGAGCTGAGCTGATAGAAGCTAACAACCAGCTAAGCCACAGGAT
                              **************************************************

MdDAM1_’Golden_delicious’     GGTGATGTATCCCAGAGGAGATATCGGACCGGAGGCCATCCTGGAGTTGG
MdDAM1_’Dorsett_Golden’       GGTGATGTATCCCAGAGGAGATATCGGACCGGAGGCCATCCTGGAGTTGG
MdDAM1-‘Anna’                 GGTGATGTATCCCAGAGGAGATATCGGACCGGAGGCCATCCTGGAGTTGG
                              **************************************************

MdDAM1_’Golden_delicious’     AAAACCTGAATAATATTGGAGAAGAAAGCATGACATCTGAATCAACCACA
MdDAM1_’Dorsett_Golden’       AAAACCTGAATAATATTGGAGAAGAAAGCGTGACATCTGAATCAACCACA
MdDAM1-‘Anna’                 AAAACCTGAATAATATTGGAGAAGAAAGCGTGACATCTGAATCAACCACA
                              *****************************.********************

MdDAM1_’Golden_delicious’     AATGTCACCACCTGCTCCAACAGCTCTCTTTCCCTTGAAGATGATTGCTC
MdDAM1_’Dorsett_Golden’       AATGTCACCACCTGCTCCAACAGCTCTCTTTCCCTTGAAGATGATTGCTC
MdDAM1-‘Anna’                 AATGTCACCACCTGCTCCAACAGCTCTCTTTCCCTTGAAGATGATTGCTC
                              **************************************************

MdDAM1_’Golden_delicious’     CGACATCTTGTCTCTCAAACTGGGGTGA
MdDAM1_’Dorsett_Golden’       CGACATCTTGTCTCTCAAACTGGGGTGA
MdDAM1-‘Anna’                 CGACATCTTGTCTCTCAAACTGGGGTGA
                              ****************************
```

B

```
MdDAM1_’Dorsett_Golden’       MKIKIKKIDYLPARQVTFSKRRRGIFKKAGELSILCESEVAVIIFSQTGK
MdDAM1-‘Anna’                 MKIKIKKIDYLPARQVTFSKRRRGIFKKAGELSILCESEVAVIIFSQTGK
MdDAM1_’Golden_delicious’     MKIKIKKIDYLPARQVTFSKRRRGIFKKAGELSILCESEVAVIIFSQTGK
                              **************************************************

MdDAM1_’Dorsett_Golden’       LFDFSSSSTKDVIARYNSHIGGEKSDQPTIHQLQLEKENNIRLRKELEDK
MdDAM1-‘Anna’                 LFDFSSSSTKDVIARYNSHIGGEKSDQPTIHQLQLEKENNIRLRKELEDK
MdDAM1_’Golden_delicious’     LFDFSSSSTKDVIARYNSHIGGEKSDQPTIHQLQLEKENNIRLRKELEDK
                              **************************************************

MdDAM1_’Dorsett_Golden’       SCKLRQMKGVDLEDLDLDELQKLEKLVEASLGRVIQTKEEKIMSEVMALE
MdDAM1-‘Anna’                 SCKLRQMKGVDLEDLDLDELQKLEKLVEASLGRVIQTKEEKIMSEVMALE
MdDAM1_’Golden_delicious’     SCKLRQMKGVDLEDLDLDELQKLEKLVEASLGRVIQTKEEKIMSEVMALE
                              **************************************************

MdDAM1_’Dorsett_Golden’       KKGAELIEANNQLSHRMVMYPRGDIGPEAILELENLNNIGEESVTSESTT
MdDAM1-‘Anna’                 KKGAELIEANNQLSHRMVMYPRGDIGPEAILELENLNNIGEESVTSESTT
MdDAM1_’Golden_delicious’     KKGAELIEANNQLSHRMVMYPRGDIGPEAILELENLNNIGEESMTSESTT
                              *******************************************:******

MdDAM1_’Dorsett_Golden’       NVTTCSNSSLSLEDDCSDILSLKLG-
MdDAM1-‘Anna’                 NVTTCSNSSLSLEDDCSDILSLKLG-
MdDAM1_’Golden_delicious’     NVTTCSNSSLSLEDDCSDILSLKLG-
                              *************************
```

Figure S2. Single nucleotide polymorphisms detected in MdDAM1 sequence of low-chill cultivars.

The nucleotide (A) and amino acid (B) sequences of *MdDAM1* from ‘Golden Delicious’ was aligned to those of the low-chilling cultivars ‘Dorsett Golden’ and ‘Anna’. The single nucleotide polymorphisms (SNPs) in (A) are highlighted in blue. The M (green) to V (blue) amino acid mutation at position 194 is indicated. The MADS domain (Interpro domain IPR002100) is highlighted in gray and the K-box domain indicated in bold characters.

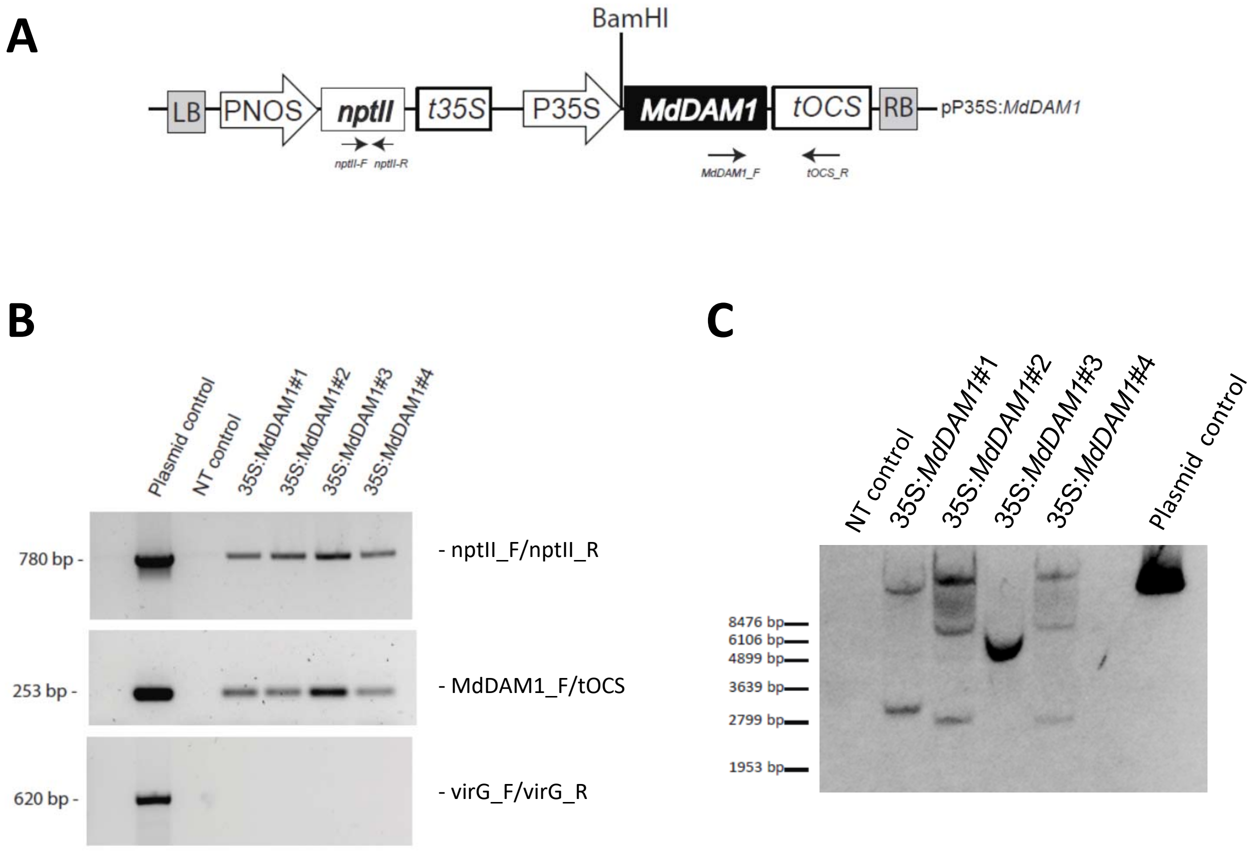

Figure S3. Molecular characterization of the 35S:*MdDAM1* transgenic lines.

Schematic representation of the pP35S:*MdDAM1* construct. The sequences of the primers shown are indicated in Table S2. The BamHI restriction site used to digest the genomic DNA for the Southern blot analysis is indicated (A). End-point PCR amplification of different regions of pP35S:*MdDAM1* construct in the transgenic lines 35S:MdDAM1#1-4 and the non-transformed control (NT control). The plasmid was used as a control for PCR amplification (B). Southern blot analysis using a *nptII* probe. The base pair (bp) ladder is indicated on the left (C).

A

```
                1                                                                                                                         130
   MdDAM1                           AT GAAGATCAAG ATAAAAAAGA TCGACTACTT GCCGGCAAGG CAGGTGACCT TCTCAAAGAG GAGAAGGGGG ATTTTCAAGA AAGCTGGAGA GCTGTCGATT CTGTGTGAAT
   MdDAM4       ATGGTGAAAA GGATGAATGA GAAGATTAAG ATCAGGAGGA TCGACTACTT GCCGGCAAGG CAGGTGACCT TCTCAAAGAG GAGAAGAGGG ATTTTCAAGA AAGCTGAAGA GCTGTCGATT CTGTGTGAAT
Consensus       .......... ........aa GAAGATcAAG ATaAaaAaGA TCGACTACTT GCCGGCAAGG CAGGTGACCT TCTCAAAGAG GAGAAGaGGG ATTTTCAAGA AAGCTGaAGA GCTGTCGATT CTGTGTGAAT

                131                                                                                                                       260
   MdDAM1       CTGAAGTTGC TGTTATCATC TTTTCTCAAA CTGGCAAGCT CTTTGATTTC TCAAGCTCCA GCACCAAGGA TGTGATTGCA AGGTACAATT CACATATCGG TGGGGAAAAA TCGGATCAAC CCACGATTCA
   MdDAM4       CTGAAGTTGC TGTTATCATC TTTTCTCAAA CTGGCAAGCT CTTTGATTAC TCAAGCACCA GTACCAAGGA TGTGATTGCA AGGTACAAAT CACATACTGG TGGGGAAAAA TCGGATCAAA TCACGCTTCA
Consensus       CTGAAGTTGC TGTTATCATC TTTTCTCAAA CTGGCAAGCT CTTTGATTaC TCAAGCaCCA GcACCAAGGA TGTGATTGCA AGGTACAAaT CACATAccGG TGGGGAAAAA TCGGATCAAa cCACGaTTCA

                261                                                                                                                       390
   MdDAM1       TCAGCTACAG TTGGAGAAAG AAAACAATAT CAGGCTGAGG AAGGAACTTG AGGATAAGAG TTGCAAGTTG AGGCAGATGA AGGGTGTGGA CCTTGAAGAC TTGGATCTGG ATGAACTACA GAAGTTAGAA
   MdDAM4       CCAACTGCAG TCGGAGAAAG AAAACACGAT CAGGCTGAGT AAGGAACTTG AGGATAAGAC CCGCAAGCTG AGGCATATGA AGGGTGAGGA CCTTCAAGAC TTGGATCTGG ATCAACTGAA CAAGTTAGAA
Consensus       cCAaCTaCAG TcGGAGAAAG AAAACAagAT CAGGCTGAGg AAGGAACTTG AGGATAAGAc ccGCAAGcTG AGGCAgATGA AGGGTGaGGA CCTTcAAGAC TTGGATCTGG ATcAACTaaA cAAGTTAGAA

                391                                                                                                                       520
   MdDAM1       AAATTGGTGG AAGCAAGCCT TGGCCGTGTG ATTCAAACTA AGGAAGAAAA GATTATGAGT GAGGTTATGG CACTTGAGAA AAAGGGAGCT GAGCTGATAG AAGCTAACAA CCAGCTAAGC CACAGGATGG
   MdDAM4       AAATTGGTGG AAGTAAGCAT TGGCCGTGTA ATAAAAACTA AGGAAAAAAA GATAATGAGT GAGATTATGG CACTTACGAA CAAGGGAGCT GAGCTTATAG AAGCTAACAA CCAACTAAAG CAGAGGTTGG
Consensus       AAATTGGTGG AAGcAAGCaT TGGCCGTGTa ATaaAAACTA AGGAAaAAAA GATaATGAGT GAGaTTATGG CACTTaaGAA aAAGGGAGCT GAGCTgATAG AAGCTAACAA CCAaCTAAac CAcAGGaTGG

                521                                                                                                                       650
   MdDAM1       TGATGTATCC CA---GAGGA GATATCGGAC CGGAGGCCAT CCTGGAGTTG GAAAACCTGA ATAATATTGG AGAAGAAAGC ATGACATCTG AATCAACCAC AAATGTCACC ACCTGCTCCA ACAGCTCTCT
   MdDAM4       TGATGTTATC CGCTGGAGGA GATATCGAAC CGGCGGCGAT CATGGAGTTG GAAAACCTGA ATAATGTTGG AGAAGAAGGC ATGACATCTG AATCAGCCAC AAATGTCACC GCCTGCTCCA GCAGTGCTCT
Consensus       TGATGTaacC Ca...GAGGA GATATCGaAC CGGaGGCcAT CaTGGAGTTG GAAAACCTGA ATAATaTTGG AGAAGAAaGC ATGACATCTG AATCAaCCAC AAATGTCACC aCCTGCTCCA aCAGcgCTCT

                651                                                705
   MdDAM1       TTCCCTTGAA GATGATTGCT CCGACATCTT GTCTCTCAAA CTGGGGTGA
   MdDAM4       TTCTCTCGAA GATGACTGCT CCGACATCTT GTCTCTCAAA CTGGGGCTAC CTTAG
Consensus       TTCcCTcGAA GATGAcTGCT CCGACATCTT GTCTCTCAAA CTGGGGcgA. .....
```

B

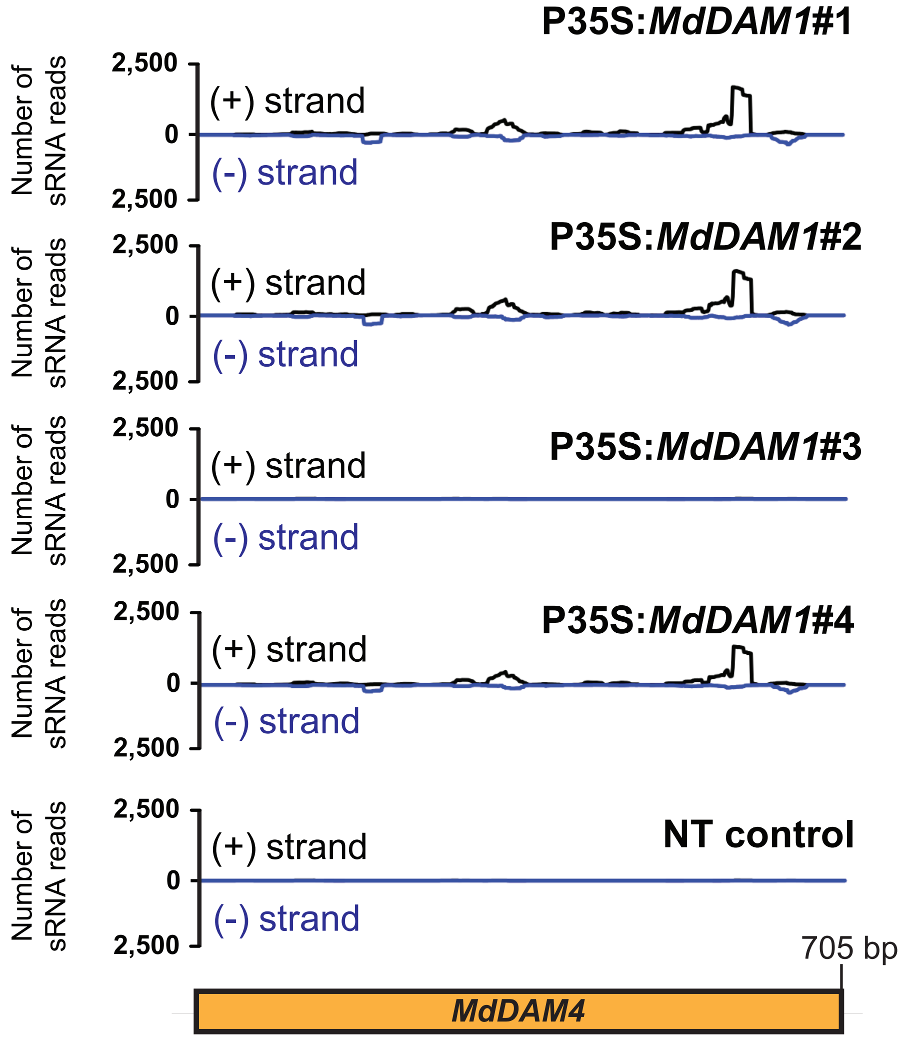

Figure S4. Similarity between *MdDAM1* and *MdDAM4* and sRNA abundance mapping on *MdDAM4*

Alignment of *MdDAM1* and *MdDAM4* shows a high similarity at the nucleotide level. Similar nucleotides are indicated in red (A). The abundance of small RNAs (sRNA) generated in 35S:*MdDAM1* transgenic lines and mapping on *MdDAM4* with a perfect match is indicated (B). The number of small RNA (sRNA) ranging from 19 nt to 24 nt mapping in sense (+ strand) or in antisense (- strand) are indicated by the black and blue lines, respectively.
The flat lines shown in the NT control and in line P35S:*MdDAM1*#3 indicate that there were no sRNAs mapping on *MdDAM4*.
